# Estrogen signaling in the dorsal raphe regulates binge-like drinking in mice

**DOI:** 10.1038/s41398-024-02821-2

**Published:** 2024-02-27

**Authors:** Valeria C. Torres Irizarry, Bing Feng, Xiaohua Yang, Nirali Patel, Sarah Schaul, Lucas Ibrahimi, Hui Ye, Pei Luo, Leslie Carrillo-Sáenz, Penghua Lai, Maya Kota, Devin Dixit, Chunmei Wang, Amy W. Lasek, Yanlin He, Pingwen Xu

**Affiliations:** 1 Division of Endocrinology, Department of Medicine, The University of Illinois at Chicago, Chicago, IL 60612 USA; 2 Department of Physiology and Biophysics, The University of Illinois at Chicago, Chicago, IL 60612 USA; 3 Pennington Biomedical Research Center, Louisiana State University, Baton Rouge, LA 70808 USA; 4 Guangdong Laboratory of Lingnan Modern Agriculture and Guangdong Province Key Laboratory of Animal Nutritional Regulation, National Engineering Research Center for Breeding Swine Industry, College of Animal Science, South China Agricultural University, 483 Wushan Road, Tianhe District, 510642 Guangzhou, Guangdong China; 5 Children’s Nutrition Research Center, Department of Pediatrics, Baylor College of Medicine, One Baylor Plaza, Houston, TX 77030 USA; 6 Center for Alcohol Research in Epigenetics and Department of Psychiatry, University of Illinois at Chicago, Chicago, IL 60612 USA; 7 Present Address: Department of Pharmacology and Toxicology, Virginia Commonwealth University, Richmond, VI 23298 USA

**Keywords:** Neuroscience, Physiology

## Abstract

Estrogens promote binge alcohol drinking and contribute to sex differences in alcohol use disorder. However, the mechanisms are largely unknown. This study aims to test if estrogens act on 5-hydroxytryptamine neurons in the dorsal raphe nucleus (5-HT^DRN^) to promote binge drinking. We found that female mice drank more alcohol than male mice in chronic drinking in the dark (DID) tests. This sex difference was associated with distinct alterations in mRNA expression of estrogen receptor α (ERα) and 5-HT-related genes in the DRN, suggesting a potential role of estrogen/ERs/5-HT signaling. In supporting this view, 5-HT^DRN^ neurons from naïve male mice had lower baseline firing activity but higher sensitivity to alcohol-induced excitation compared to 5-HT^DRN^ neurons from naïve female mice. Notably, this higher sensitivity was blunted by 17β-estradiol treatment in males, indicating an estrogen-dependent mechanism. We further showed that both ERα and ERβ are expressed in 5-HT^DRN^ neurons, whereas ERα agonist depolarizes and ERβ agonist hyperpolarizes 5-HT^DRN^ neurons. Notably, both treatments blocked the stimulatory effects of alcohol on 5-HT^DRN^ neurons in males, even though they have antagonistic effects on the activity dynamics. These results suggest that ERs’ inhibitory effects on ethanol-induced burst firing of 5-HT^DRN^ neurons may contribute to higher levels of binge drinking in females. Consistently, chemogenetic activation of ERα- or ERβ-expressing neurons in the DRN reduced binge alcohol drinking. These results support a model in which estrogens act on ERα/β to prevent alcohol-induced activation of 5-HT^DRN^ neurons, which in return leads to higher binge alcohol drinking.

## Introduction

Binge drinking, defined as the consumption of a significant amount of alcohol in a single setting [1, 2], is the most common pattern of excessive alcohol use in the US [3] and contributes to the development of alcohol use disorder (AUD). Like many other psychiatric disorders, there are sex differences in the trajectory of AUD and its consequences. Binge drinking by women has increased faster than by men in recent decades [4] and women show more vulnerability to alcohol-induced cognitive impairment and peripheral neuropathy than men [5–9]. While more effective treatments to reduce alcohol misuse in both sexes are urgently needed, our understanding of the pathophysiology of female binge drinking is limited.

A large body of evidence indicates that the ovarian hormone estrogens play a role in sex differences in alcohol consumption behavior. Human clinical studies support the association between increased estrogen levels and increased alcohol use [10–13]. Consistently, numerous animal studies have demonstrated the stimulatory effects of 17β-estradiol (E2) on ethanol drinking under various access conditions, including those that promote binge drinking [14–19]. Overall, human and animal studies implicate estrogens in increased alcohol drinking. However, little is known about the central mechanisms mediating estrogenic regulation of behaviors related to alcohol drinking. Only a handful of studies have shown that ERs in the ventral tegmental area mediate estrogen’s effects on the dopamine system and increase alcohol binge-like drinking behavior in female mice [20–22]. Nevertheless, the other potential ER sites in the brain mediating estrogenic effects on alcohol binge drinking have not been identified.

The brain serotonin (5-hydroxytryptamine, 5-HT) system is a critical modular of alcohol intake and is critically involved in alcohol’s effects on the brain and the development of alcohol misuse [23–25]. It has been reported that binge drinking induces a burst release of central 5-HT, and increased brain 5-HT content inhibits alcohol consumption in both humans and rodents [23–26]. Interestingly, estrogens have a potent modulatory impact on this system. For example, estrogens have been shown to increase serotonergic tone by regulating the synthesis and degradation of serotonin [27–29]. In line with these findings, both estrogen receptor α (ERα) and β (ERβ) are highly expressed in the dorsal raphe (DRN) [30], the largest serotonergic nucleus and a major source of 5-HT. It has been shown that the ERα agonist propyl pyrazole triol (PPT) increases [31, 32], whereas the ERβ agonist diarylpropionitrile (DPN) decreases the spontaneous firing of the serotonergic neurons in the DRN [33]. These studies demonstrate a potential mediating role of DRN 5-HT neurons (5-HT^DRN^) in the regulatory effects of estrogen on binge drinking.

In this study, we first used chronic drinking in the dark (DID) behavioral test to examine the effects of long-term binge drinking on estrogen and 5-HT signaling in the DRN. Furthermore, the ex vivo responsiveness of 5-HT^DRN^ neurons to ethanol treatment was compared between male and female mice using whole-cell patch-clamp electrophysiology recording. We further tested whether E2, PPT, and DPN treatments attenuate the sex difference in ethanol-induced activity changes of 5-HT^DRN^ neurons. Finally, we used the designer receptors exclusively activated by designer drugs (DREADD) approach to examine the effects of DRN neural activity on binge drinking in mice. Our results provide compelling evidence to support a model in which estrogens act on ERα/β to prevent alcohol-induced activation of 5-HT^DRN^ neurons that inhibit binge drinking.

## Results

### Sex-specific gene changes in the DRN induced by chronic DID

**Fig. 1:**
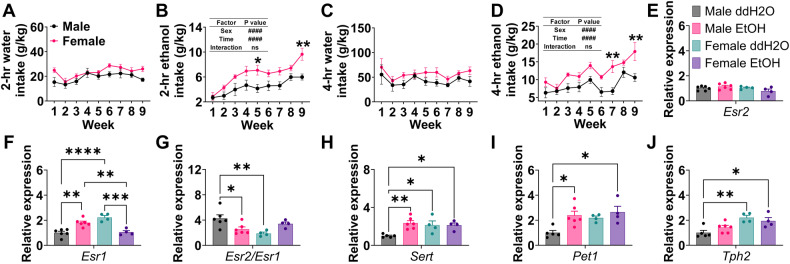
Chronic binge-like ethanol drinking leads to sex-specific alterations in Esr1 and 5-HT-related genes in the DRN of mice. **A** and **B** Average water (**A**) or EtOH (**B**) consumed during 2-h drinking sessions on days 1–4 each week (*n* = 5 males or 7 females). **C** and **D** Average water (**C**) or EtOH (**D**) consumed during 4-h drinking sessions on day 4 each week (*n* = 5 males or 7 females). **E**–**J** Relative mRNA expression of estrogen receptor 2 (*Esr2*, **E**), estrogen receptor 1 (*Esr1*, **F**), *Esr1*/*Esr2* (**G**), serotonin transporter (*Sert*, **H**), plasmacytoma expressed transcript 1 (*Pet1*, **I**), and tryptophan hydroxylase 2 (*Tph2*, **J**) in the DRN (*n* = 6 males or 4 females). Mean ± SEM. **A**–**D** ####*p* < 0.0001 in two-way ANOVA analysis, *p < 0.05, **p < 0.01 in the following post hoc Sidak tests. **E**–**J** *p < 0.05, **p < 0.01, ***p < 0.001, ****p < 0.0001 in one-way ANOVA analysis followed by post hoc Tukey tests.

Both male and female C57BL/6J mice were subjected to 9-week water or ethanol DID. We found females consumed more alcohol than males, but not water in the DID test (Fig. 1A–D). Notably, 9-week binge-like ethanol drinking leads to increases in the mRNA expression of estrogen receptor 1 (*Esr1*, the gene encoding ERα protein), serotonin transporter (*Sert*, serotonin reuptake enzyme), and plasmacytoma expressed transcript 1 (*Pet1*, a crucial transcription factor for the metabolism and the reuptake of serotonin) in males but not females. Conversely, chronic binge-like drinking decreased the mRNA expression of *Esr1* but not 5-HT-related genes in females (Fig. 1E–J), suggesting a sexually dimorphic response in the DRN to chronic binge-like ethanol drinking.

Notably, we found that average 2-h alcohol consumption negatively correlated with *Pet1* mRNA in the DRN (*R* = −0.62, *P* = 0.05, Fig. S1). Additionally, *Tph2* mRNA showed a negative correlation with *Esr2* mRNA in the DRN (*R* = −0.80, *P* = 0.006, Fig. S1). These results further support a link between alcohol-drinking behaviors, estrogen signaling, and the serotonergic system in the DRN.

### 5-HT^DRN^ neurons co-express ERα/ERβ

**Fig. 2:**
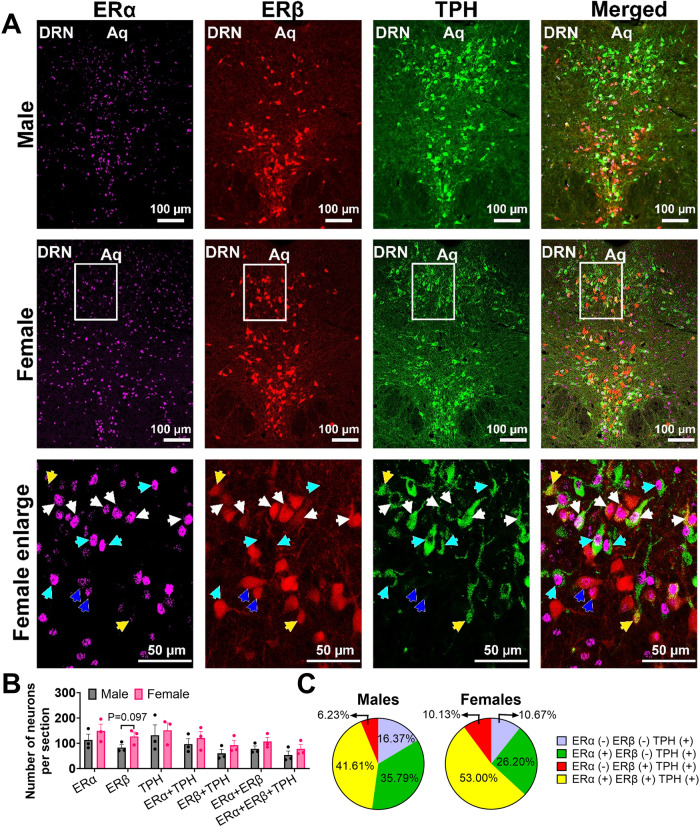
ERα&β are expressed in 5-HT^DRN^ neurons. **A** Double immunofluorescent staining for ERα (purple) and TPH (green) in the DRN of *Esr2*-Cre/Rosa26-tdTOMATO mice. The white square represents an enlarged area. White arrows point to triple-colored neurons. Yellow arrows point to dual-colored neurons with ERβ (red) and TPH (green) expressed. Blue arrows point to dual-colored neurons with ERα (purple) and ERβ (red) expressed. Cyan arrows point to dual-colored neurons with ERα (purple) and TPH (green) expressed. **B** Summary of quantification per section (*n* = 3 and 3). **C** Percentage of ERα and ERβ positivity in TPH^DRN^ neurons. Mean ± SEM.

To examine the expression pattern of ERα and ERβ on serotonergic neurons in the DRN, we did dual immunofluorescent (IF) staining of ERα and tryptophan hydroxylase (TPH) in the *Esr2*-Cre/Rosa26-LSL-tdTOMATO mice, in which all ERβ positive neurons were labeled with red TOMATO fluorescence. We found that about 80–90% of 5-HT^DRN^ neurons co-express ERα or ERβ (83.63% in M vs. 89.33% in F); about 40–50% of 5-HT^DRN^ neurons are positive for both ERα and ERβ (41.61% in M vs. 53% in F, Fig. 2A–C). Notably, there are more ERα (+) 5-HT^DRN^ neurons than ERβ (+) 5-HT^DRN^ neurons in both males and females (77.4% vs. 47.84% in M; 79.2% vs. 63.13% in F, Fig. 2C). We also found that around 80% of ERα^DRN^ neurons (85.28% in M vs. 79.48% in F) and 70% of ERβ^DRN^ neurons (70.26% in M vs. 73.02% in F; Fig. S2A, B) are serotonergic. Consistently, we found abundant ERα and ERβ expression in 5-HT neurons in the DRN of ERβ-EGFP mice, as indicated by white arrow-pointed triple color-positive neurons (Fig. S2C–K). These provide the anatomic basis that estrogen acts through both ERα/β expressed by 5-HT^DRN^ neurons to regulate binge drinking behavior.

### Sexually dimorphic responses to ethanol in ERα^DRN^ neurons

**Fig. 3:**
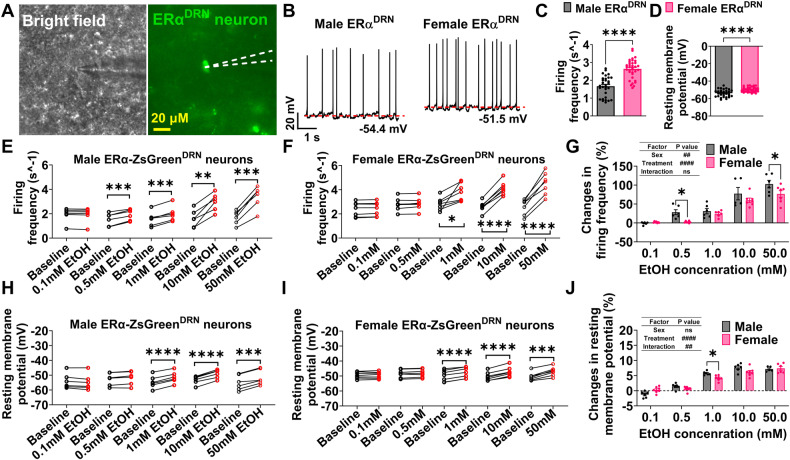
Sex difference in the responses of ERα^DRN^ neurons to ethanol. **A** Micrographic images showing recorded ERα-ZsGreen (+) neurons in the DRN of female mice. **B**–**D** Representative electrophysiological trace (**B**), baseline firing frequency (**C**), and resting membrane potential (**D**) of ERα-ZsGreen (+) neurons in the DRN of male and female ERα-ZsGreen mice (*n* = 30 in male or 35 in female). **E**–**J** The dose-dependent responses of firing frequency and resting membrane potential (*n* = 6 in male or 7 in female) before and after a 1 s puff of aCSF containing different doses of EtOH. Mean ± SEM. **C** and **D** ****p < 0.0001 in unpaired *t*-tests. **E**, **F** and **H**, **I** *p < 0.05, **p < 0.01, ***p < 0.001, ****p < 0.0001 in paired *t*-tests. **G** and **J** ##*p* < 0.01 in two-way ANOVA analysis, *p < 0.05 in the following post hoc Sidak tests.

To determine the baseline characteristics of ERα^DRN^ neurons, we recorded DRN ZsGreen(+) neurons in ex vivo brain slices from ERα-ZsGreen mice (Fig. 3A). We found that ERα^DRN^ neurons from female mice showed higher firing frequency and depolarized resting membrane potential compared to ERα^DRN^ neurons from male mice (Fig. 3B–D), indicating a higher spontaneous neural activity in ERα^DRN^ neurons. We further tested the dose responses of ERα^DRN^ neurons to ethanol puff treatment while blocking the synaptic input from upstream neurons (preincubation with 30 µM CNQX + 30 µM D-AP5 + 50 µM bicuculline). This allowed us to assess the direct effects of ethanol. ERα^DRN^ neurons from males were more sensitive to ethanol-induced excitation compared to ERα^DRN^ neurons from females (Fig. 3E–J). Specifically, while a low dose of 0.5 mM ethanol treatment failed to increase the firing frequency of female ERα^DRN^ neurons (Fig. 3F), it significantly raised the firing rate of male ERα^DRN^ neurons (Fig. 3E). Consistently, the ethanol-induced increases in firing frequency were substantially more significant in males than in females at 0.5 and 50 mM doses (Fig. 3G). The changes in resting membrane potential were also greater in males at a dose of 1 mM ethanol treatment compared to female mice (Fig. 3H–J). Since a majority of ERα-expressing cells within the DRN are 5-HT neurons (Fig. 2D) [32], we speculate that ERα-expressing 5-HT^DRN^ neurons exhibit a sexually dimorphic response to ethanol treatment.

### Estrogen attenuates ethanol-induced excitation of 5-HT^DRN^ neurons in males

**Fig. 4:**
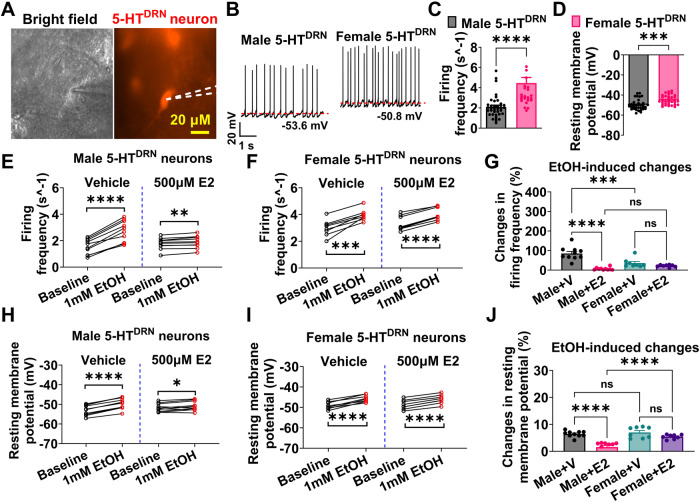
Estrogen attenuates EtOH-induced activation of 5-HT^DRN^ neurons. **A** Micrographic images showing a recorded TPH2 (+) neuron in the DRN of female TPH2-CreER/Rosa26-tdTOMATO mice. **B**–**D** Representative electrophysiological trace (**B**), baseline firing frequency (**C**), and resting membrane potential (**D**) of TPH2 (+) neurons in the DRN of male and female mice (*n* = 38 in male or 26 in female). **E**–**J** The responses of firing frequency and resting membrane potential to EtOH treatment in the presence of vehicle or 500 µM 17β-Estradiol (*n* = 10 in male or 8 in female). Mean ± SEM. **C** and **D** ***p < 0.001, ****p < 0.0001 in unpaired *t*-tests. **E**, **F** and **H**, **I** *p < 0.05, **p < 0.01, ***p < 0.001, ****p < 0.0001 in paired *t*-tests. **G** and **J** ***p < 0.001, ****p < 0.0001 in one-way ANOVA analysis followed by post hoc Tukey tests.

To compare the baseline characteristics of 5-HT^DRN^ neurons from different sexes, we recorded DRN tdTOMATO (+) neurons in ex vivo brain slices from TPH2-iCreER/Rosa26-LSL-tdTOMATO mice (Fig. 4A). Similar to what we observed in ERα^DRN^ neurons, female 5-HT^DRN^ neurons had higher spontaneous activity, as indicated by increased firing frequency and depolarized resting membrane potential (Fig. 4B–D). To further test if estrogens contribute to sex differences in ethanol’s effects on DRN neuron firing, we recorded the responses of 5-HT^DRN^ neurons to 1 s puff treatment of 1 mM ethanol in the presence of vehicle or E2 after blocking glutamatergic and GABAergic neurotransmission. Consistent with ethanol’s dose-dependent stimulatory effects on ERα^DRN^ neurons (Fig. 3E–J), 1 mM ethanol puff significantly increased firing frequency and depolarized resting membrane potential of 5-HT^DRN^ neurons regardless of sex or E2 treatment (Figs. 4E, F and H, I; S3A, B). Notably, the ethanol-induced increases in firing frequency and resting membrane potentials were significantly reduced in males but not in females by E2 treatment (Fig. 4G and J), suggesting an attenuation in ethanol-induced activation of 5-HT^DRN^ neurons.

### ERα agonist stimulates ERα^DRN^ neurons while ERβ agonist inhibits 5-HT^DRN^ neurons

**Fig. 5:**
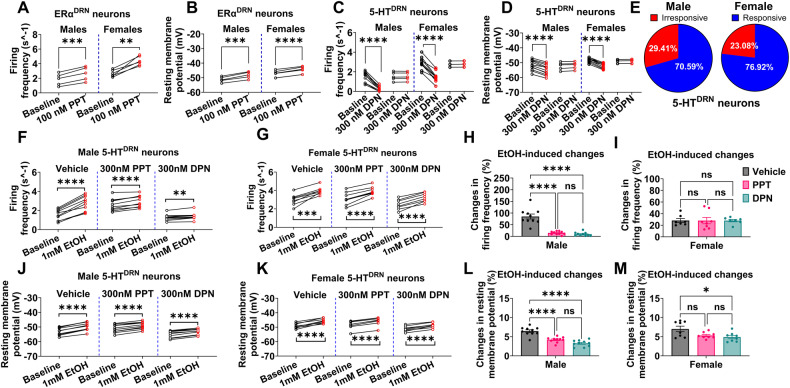
A selective agonist for ERα or ERβ attenuates EtOH-induced activation of 5-HT^DRN^ neurons. **A** and **B** The firing frequency (**A**) and resting membrane potential (**B**) of ERα (+) neurons in the DRN of ERα-ZsGreen mice before and after a 1 s puff of aCSF containing 100 nM PPT (*n* = 5 in male or 6 in female). **C**–**E** The firing frequency (**C**), resting membrane potential (**D**), and responsive rate (**E**) of TPH2 (+) neurons in the DRN of TPH2-CreER/Rosa26-tdTOMATO mice before and after a 1 s puff of aCSF containing 100 nM DPN (*n* = 11 in male or 10 in female). **F**–**M** The responses of firing frequency and resting membrane potential to EtOH treatment in the presence of vehicle, 300 nM PPT, or 300 nM DPN in TPH2 (+) neurons in the DRN of male and female TPH2-CreER/Rosa26-tdTOMATO mice (*n* = 10 in male or 10 in female). Mean ± SEM. **A**–**D**, **F**, **G**, and **J**, **K** **p < 0.01, ***p < 0.001, ****p < 0.0001 in paired *t*-tests. **H**, **I** and **L**, **M** *p < 0.05, ****p < 0.0001 in one-way ANOVA analysis followed by post hoc Tukey tests within each sex.

To explore the intracellular mechanism for estrogenic action on 5-HT^DRN^ neurons, we recorded the responses of ERα^DRN^ or 5-HT^DRN^ neurons to treatment with the ERα agonist, propyl pyrazole triol (PPT), or the ERβ agonist, diarylpropionitrile (DPN), respectively. In both male and female mice, we found that PPT significantly increased firing frequency and depolarized resting membrane potential of ERα^DRN^ neurons (Figs. 5A, B; S4A, B), which is consistent with our previous report on the ERα-mediated stimulatory effects of PPT on 5-HT^DRN^ neurons [32]. This is also in line with the observations that most ERα-expressing neurons are 5-HT positive neurons (Fig. S2A). Conversely, DPN significantly decreased the firing frequency and hyperpolarized resting membrane potential of 5-HT^DRN^ neurons (Figs. 5C, D; S4C, D), suggesting ERβ-mediated inhibition on 5-HT^DRN^ neurons [33]. Notably, around 70–77% of 5-HT^DRN^ neurons responded to DPN treatment (Fig. 5E), consistent with the observation that 70–73% of 5-HT^DRN^ neurons co-express ERβ (Fig. 2C). These results suggest antagonistic roles for ERα and ERβ expressed by 5-HT^DRN^ neurons in neural activity regulation.

### Both ERα and ERβ agonists attenuate EtOH-induced excitation of 5-HT^DRN^ neurons

To identify the primary mediating receptor for E2’s inhibitory effects on ethanol-induced activation of 5-HT^DRN^ neurons, we pre-incubated DRN-containing brain slices from TPH2-CreER/Rosa26-tdTOMATO mice with either PPT or DPN. Subsequently, we tested the response of 5-HT^DRN^ neurons to ethanol puff treatment after blocking glutamatergic and GABAergic neurotransmission. We found that 1 mM ethanol puff significantly increased the firing frequency and depolarized resting membrane potential in 5-HT^DRN^ neurons regardless of sex or agonist treatment (Fig. 5F, G and J, K). Importantly, the 5-HT^DRN^ neurons from males showed attenuated responses to ethanol in the presence of PPT or DPN (Fig. 5H and L). Conversely, in females, only DPN showed inhibitory effects on ethanol-induced changes in resting membrane potential (Fig. 5I and M). These results suggest that both ERα and ERβ contribute to E2’s inhibitory effects on ethanol-induced activation of 5-HT^DRN^ neurons.

### Chemogenetic activation of ERα^DRN^ or ERβ^DRN^ neurons attenuates binge drinking in females

**Fig. 6:**
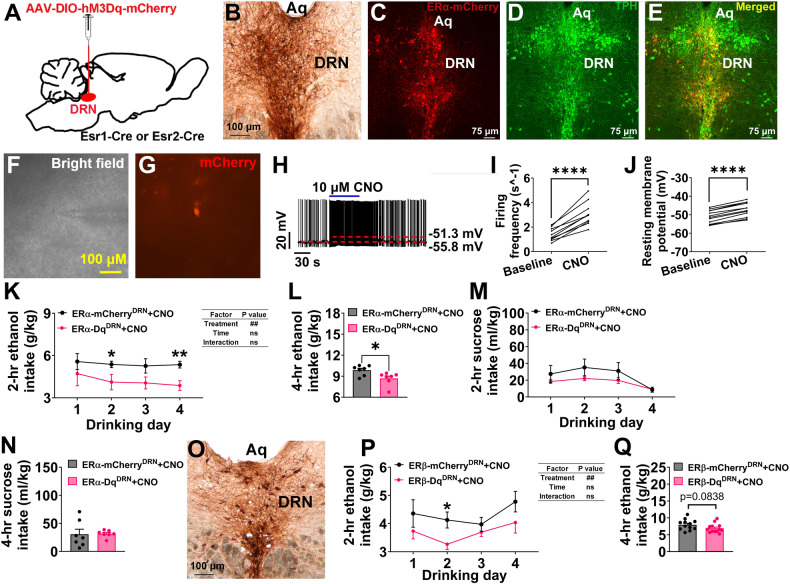
Activation of ERα^DRN^ or ERβ^DRN^ neurons decreases ethanol drinking. **A** Schematic of the experimental strategy using the AAV-DIO-hM3Dq-mCherry virus to selectively activate ERα^DRN^ or ERβ^DRN^ neurons in *Esr1*-Cre or *Ers2*-Cre female mice (ERα-Dq^DRN^ or ERβ-Dq^DRN^); *Esr1*-Cre or *Esr2*-Cre mice receiving the AAV-DIO-mCherry virus injections were used as controls (ERα-mCherry^DRN^ or ERβ-mCherry^DRN^). **B** Immunostaining of ERα-hM3Dq-mCherry in the DRN of female ERα-Dq^DRN^ mice. **C**–**E** Immunofluorescence staining of ERα-hM3Dq-mCherry (**C**), TPH (**D**), and merger (**E**) in the DRN of female ERα-Dq^DRN^ mice. **F** and **G** Micrographic images showing recorded ERα-hM3Dq-mCherry (+) neurons in the DRN. **H**–**J** Representative traces and statistical analysis before and after CNO treatment (*n* = 12). **K** and **L** Ethanol intake in g/kg over 2 h during 4 days of drinking (**K**) or during the final 4-hrs drinking session (**L**) after i.p. injection of CNO in female ERα-Dq^DRN^ or ERα-mCherry^DRN^ mice (*n* = 7). **M** and **N** Sucrose intake in ml/kg over 2 h during 4 days of drinking (**M**) or during the final 4-h drinking session (**N**) after i.p. injection of CNO in female ERα-Dq^DRN^ or ERα-mCherry^DRN^ mice (*n* = 7). **O** Immunostaining of ERβ-hM3Dq-mCherry in the DRN of female ERβ-Dq^DRN^ mice. **P** and **Q** Ethanol intake over 2 h during 4 days of drinking (**P**) or during the final 4-h drinking session (**Q**) after i.p. injection of CNO in female ERβ-Dq^DRN^ (*n* = 12) or ERβ-mCherry^DRN^ mice (*n* = 13). Mean ± SEM. **I** and **J** ****p < 0.0001 in paired *t*-tests. **K** and **P** ##*p* < 0.01 in two-way ANOVA analyses; *p < 0.05, **p < 0.01 in the following post hoc Bonferroni tests. **L** *p < 0.05 in non-paired *t*-tests.

To directly test the regulatory effects of ERα^DRN^ neurons on binge drinking, we employed the DREADD method to selectively activate ERα^DRN^ neurons by stereotaxic injection of AAV-DIO-hM3Dq-mCherry into the DRN of *Esr1*-Cre mice (ERα-Dq^DRN^, Fig. 6A). To exclude the possible off-target effects of clozapine N-oxide (CNO) or its metabolites [34], we included *Esr1*-Cre mice receiving the AAV-DIO-mCherry virus injections as controls (ERα-mCherry^DRN^). We observed specific expression of hM3Dq-mCherry in female ERα-Dq^DRN^ mice (Fig. 6B). Notably, the mCherry-positive neurons in the DRN were identified to be TPH neurons (Fig. 6C-E), and CNO treatment increased firing frequency and resting membrane potential of these neurons (Fig. 6F-J), validating the successful activation of ERα-expressing 5-HT^DRN^ neurons. We found that CNO-induced activation of ERα^DRN^ neurons reduced alcohol consumption in both 2- and 4-h binge drinking sessions (Fig. 6K, L), suggesting an essential role of the ERα → 5-HT pathway in binge drinking.

To determine whether the effects of activation of ERα^DRN^ neurons on binge-like drinking were specific to ethanol or might extend to other rewarding substances, we performed the DID test using 2% sucrose (instead of ethanol). There were no significant effects of ERα^DRN^ activation for sucrose consumption during the 2-hour sessions or during the final 4-h session (Fig. 6M, N), demonstrating that activation of ERα^DRN^ neurons in female mice does not affect sucrose-induced reward effects. Finally, to determine whether the inhibitory effect of ERα^DRN^ activation on alcohol consumption is ERα-specific, we injected AAV-DIO-hM3Dq-mCherry into the DRN of *Esr2*-Cre mice (Fig. 6O) and tested the effects of ERβ^DRN^ activation on the ethanol drinking in the DID test. We found that activation of ERβ^DRN^ neurons in female mice also significantly decreased alcohol consumption during the 2-h sessions and tended to reduce ethanol drinking during the final 4-h session (Fig. 6P and Q). These results suggest a regulatory role for ERα- and ERβ-expression neurons in the DRN in ethanol binge drinking.

## Discussion

Our findings demonstrate a regulatory role of estrogen/ERs/5-HT^DRN^ signaling in binge drinking. To our knowledge, this is the first report regarding the role of estrogenic serotonin system in controlling alcohol binge drinking. The essential function of this signaling is first supported by the sex-specific alterations in mRNA expression of ERα and 5-HT-related genes in the DRN induced by chronic alcohol DID tests. We further provided ex vivo patch-clamp evidence that ethanol sex dimorphically depolarizes 5-HT^DRN^ neurons and that pharmacological activation of ERα or ERβ attenuates ethanol-induced activation of 5-HT neurons. Finally, we showed that DREADD stimulation of ERα^DRN^ or ERβ^DRN^ neurons reduces binge-like ethanol drinking. These findings implicate a potential role of estrogen signaling in 5-HT^DRN^ neurons in sex dimorphism in binge-like alcohol drinking.

The present study used a 9-week chronic DID test to study the neuroplastic alterations in the serotonergic system. It has been demonstrated that a history of binge-like alcohol drinking increases the subsequent voluntary alcohol intake in mice [35, 36], suggesting a potential to use chronic DID as a model to study the transition to an alcohol-dependence-like state [37]. Interestingly, these stimulatory effects increase with the number of binge-like drinking episodes. Excessive alcohol drinking stemming from DID procedures becomes most robust after 6–10 DID episodes but is absent in mice that experienced only 1 DID episode [38]. Based on these findings, we chose 9-week DID episodes to induce the early stages of alcohol dependence and study the sex-specific responses of the DRN serotonergic system.

Notably, the estrous cycle is an important factor that affects many psychiatric behaviors. However, studies have consistently shown that the stage of the estrous cycle does not affect alcohol-drinking behavior. For example, in mice, ethanol intake in the DID procedure is not altered by the estrous cycle, and long-term DID does not affect the estrous cycle either [16]. Additionally, the phase of the estrous cycle does not affect baseline ethanol intake in the two-bottle choice test [39]. Similar observations have been made in rats, where the estrous cycle had no effect on alcohol drinking in Long Evans or Wistar rats, whether through continuous voluntary drinking (two-bottle choice), intermittent voluntary drinking (two-bottle choice), or operant alcohol self-administration and alcohol vapor exposure [40, 41]. Therefore, it is unlikely that the female estrous cycle plays a role in female alcohol binge-drinking behavior in rodents.

Although many studies have shown an association between estrogen signaling and the brain’s 5-HT system, a key modulator of alcohol intake [23–25], the potential regulatory role of estrogenic 5-HT signaling in alcohol drinking has not been thoroughly studied. Anatomically, ERα and ERβ are expressed in the 5-HT^DRN^ neurons of male and female mice [30]. We further quantified the expression pattern of ERα, ERβ, and TPH in the DRN and found most 5-HT^DRN^ neurons are positive for ERα or ERβ. Notably, a large portion of the 5-HT^DRN^ neurons co-express both ERα and ERβ. This drove us to separately test the electrophysiological function of ERα or ERβ expressed by 5-HT^DRN^ neurons. In line with our previous reports [32, 33], we found that estrogens stimulate 5-HT^DRN^ neurons through ERα while inhibiting 5-HT^DRN^ neurons through ERβ, suggesting antagonistic effects of ERα and ERβ expressed by 5-HT^DRN^ neurons. It is puzzling that two receptors with opposite regulatory effects on neural activity are expressed in the same neurons. Interestingly, several previous studies have consistently observed that neurons in different brain regions, including the medial amygdala (MeA), the bed nucleus of the stria terminalis (BNST), and the preoptic area of the hypothalamus (POAH), co-express both ERα and ERβ [42, 43]. It has been proposed that the presence of both ERs may allow the neurons to accommodate different physiological/pathological conditions by changing the ERα/ERβ ratio and responding differentially to the actions of estrogen [43].

Consistent with this point of view, in male mice, expression of *Esr1* was significantly increased by chronic alcohol DID, resulting in a much lower *Esr2*/*Esr1* ratio in the DRN. This alcohol-induced lower ratio was associated with higher 5-HT synthesis and reuptake, as indicated by higher mRNA expression of *Sert* and *Pet1* in the DRN of males induced by chronic alcohol DID. These findings suggest that during the transition from alcohol-naive to chronic alcohol DID condition, estrogens/ERα-mediated activation may override estrogens/ERβ-mediated inhibition in 5-HT^DRN^ neurons, contributing to the higher 5-HT transmission induced by chronic alcohol DID in males.

Notably, aromatase, the key enzyme for estrogen synthesis, is highly expressed in several male brain regions, including MeA, BNST, and POAH [44]. These brain regions could project and release estrogen in the DRN. Aromatase can transform testosterone into E2, which means that even in the male brain, the DRN could be exposed to high levels of E2 produced locally from testosterone. Consistently, it has been shown that 5-HT secretion from 5-HT^DRN^ neurons in males is remarkably decreased by aromatase inhibition [45]. Male estrogen signaling may play an essential role in the serotonergic adaptation induced by chronic alcohol DID in males.

Another interesting observation is the sex difference in the *Esr2*/*Esr1* ratio in the DRN of alcohol-naïve mice. Compared to males, female naïve mice have a much lower *Esr2*/*Esr1* ratio and higher mRNA expression of key enzymes for 5-HT synthesis and reuptake in the DRN. Consistently, we also observe sex differences in the intrinsic electrophysiological properties of 5-HT^DRN^ neurons in the baseline condition without alcohol exposure. Female 5-HT^DRN^ neurons showed a much higher firing rate and depolarized resting membrane potential, indicating a higher baseline activity in female 5-HT^DRN^ neurons compared to that in males. After chronic alcohol DID, the difference in *Esr2*/*Esr1* ratio between males and females was diminished, associated with abolished sex differences in mRNA expression of *Sert*, *Pet1*, and *Tph2*. It is worth noting that, during the chronic DID test, females consume significantly more alcohol than males. Therefore, any observed differences in mRNA expression between male and female mice may not necessarily reflect true sex differences but rather may be due to the fact that females were exposed to significantly more ethanol than males. Nonetheless, our data support the idea that the dynamic expression of ERs and serotonin signaling in DRN may play a physiological role in regulating chronic alcohol DID exposure.

Prior studies dating back to the 1990s have shown that injecting ethanol systemically in vivo or applying it in a bath in an ex vivo brain slice recording reduces the firing rate of 5-HT^DRN^ neurons by increasing spontaneous GABAergic inhibition in the DRN [46–49]. However, our study observed a stimulatory effect on 5-HT^DRN^ neurons induced by acute alcohol puff treatment, which contradicts these findings. It is important to note that in our studies, puff treatment was performed while blocking the synaptic input from upstream neurons (preincubation with 30 µM CNQX + 30 µM D-AP5 + 50 µM bicuculline). This allowed us to directly assess the effects of ethanol on 5-HT neurons. In contrast, previously reported bath treatment results include both direct and indirect actions. Notably, it has been reported that acute alcohol exposure enhances 5-HT neurotransmission and synaptic release [48, 50], despite decreasing the firing rate [47] or excitability [46] of 5-HT neurons. These findings suggest a dissociation between the effects of acute ethanol administration on 5-HT neuronal activity and 5-HT synaptic activity. Our current findings may provide a potential explanation for this dissociation. Specifically, alcohol rapidly and directly stimulates 5-HT^DRN^ neurons. Additionally, it indirectly and gradually inhibits 5-HT^DRN^ neurons by enhancing presynaptic GABAergic input. The increased 5-HT synaptic release may be attributed to the rapid direct effects of alcohol on 5-HT^DRN^ neural activity. This immediate activation is then followed by subsequent inhibition caused by the enhancement of indirect presynaptic GABAergic input.

It has been previously shown that acute binge drinking causes a burst release of central 5-HT, and increased brain 5-HT inhibits alcohol consumption in humans and rodents [23–26]. These findings suggest that the alcohol-induced activation of 5-HT neurons may be an essential component of a negative feedback loop to decrease acute alcohol consumption. We speculate that the rapid responses of 5-HT^DRN^ neurons to alcohol are distinct in males and females. These differences may contribute to the sex dimorphism in the alcohol binge-like drinking behavior. To test this hypothesis, we compared the acute electrophysiological response of male and female ERα^DRN^ neurons to ethanol treatment. Consistent with our previous findings [32], we confirm that most ERα-expressing cells within the DRN are 5-HT neurons. Like 5-HT^DRN^ neurons, female ERα^DRN^ neurons also showed higher baseline neuronal firing activity dynamics than male ERα^DRN^ neurons. Importantly, we found that male ERα^DRN^ neurons showed a lower threshold responding dose (0.5 mM in males vs. 1 mM in females) and higher percentage changes in firing frequency and resting membrane potential when incubated with the same amount of ethanol. These results suggest that male ERα^DRN^ neurons are more sensitive to ethanol treatment compared to female ERα^DRN^ neurons. We postulate that lower baseline neuronal firing activity of male ERα^DRN^ neurons leads to higher burst increases of 5-HT release, contributing to earlier ethanol drinking termination.

Clear dose-response of ethanol has been observed in ERα^DRN^ neurons from both male and female mice. The lowest dose (0.1 mM ethanol puff) had little effect on resting membrane potential and firing frequency. In contrast, concentrations of 1 mM and higher up to 50 mM significantly stimulated resting membrane potential and firing frequency. Notably, the blood ethanol concentration (BEC) that is the legal limit for driving in most US states is 0.08% (80 mg/dL or 17 mM), while a BEC of 0.2% (200 mg/dL or 50 mM) is associated with moderate to severe intoxication in nontolerant humans [51]. The BEC observed in mice during an acute or chronic DID alcohol test falls in a range of 100–150 mg/dL (22–32 mM) [35, 52]. These BECs are much higher than 1 mM, the lowest ex vivo dose that induced significant changes. Notably, the BEC does not represent the actual ethanol dose in the brain [53]. Few existing studies in living humans and rats have shown that BEC is unsuitable for assessing brain ethanol content [54–57]. Additionally, we applied ethanol through puff application, which allows for localized application of ethanol with the accuracy of targeting a single neuron. A much lower dose of ethanol could achieve the same regulatory effects induced by bath application.

Sex differences in the 5-HT system and the regulatory effects of estrogens on 5-HT neuron activity have been demonstrated for decades [31, 58–60]. It has been shown that estrogen-mediated sex differences in the 5-HT system contribute to the greater susceptibility of women to many affective behavior disorders, such as premenstrual syndrome, postpartum depression, and postmenopausal depression [31, 59]. We speculate that estrogen signaling may partially mediate the sex difference in ethanol-induced excitation of 5-HT^DRN^ neurons. In supporting this view, we observed that E2 treatment significantly attenuated the ethanol-induced excitation of 5-HT^DRN^ neurons in males but not in females, suggesting an estrogen-mediated attenuation of ethanol-induced 5-HT burst release. We further demonstrated that both ERα and ERβ agonists consistently produced similar inhibitory effects on ethanol-induced firing activity changes of 5-HT^DRN^ neurons, despite antagonistic effects on activity dynamics of 5-HT^DRN^ neurons. These results suggest distinct mechanisms for ERs’ effects on baseline activity and ethanol-induced burst firing. These findings support the notion that estrogens acutely increase alcohol binge drinking by reducing the responsiveness of 5-HT neurons to ethanol.

Interestingly, we found that the majority of ERβ^DRN^ neurons express ERα (93.79% vs. 6.21% in males; 84.64% vs. 15.36% in females). Similarly, although to a lesser extent, most ERα^DRN^ neurons also express ERβ (70.72% vs. 29.28% in males; 73.38% vs. 26.62% in females). These anatomical colocalizations suggest potential crosstalk between ERα and ERβ intracellular signaling in the same neurons. This is especially significant considering that these two receptors mediate opposite acute electrophysiological responses, with ERα inducing depolarization and ERβ inducing hyperpolarization. Nevertheless, both ERα and ERβ agonists seem to exert the same inhibitory effects on the alcohol-induced burst firing of 5-HT^DRN^ neurons. This suggests a synergistic inhibitory effect mediated by both receptors to prevent alcohol-induced activation and subsequent release of 5-HT, potentially disrupting an acute protective mechanism against alcohol overconsumption.

Of note, pharmacological activation of ERα or ERβ acutely modulated the resting membrane potential and firing rate of 5-HT^DRN^ neurons, which is much faster than the slow transcriptional regulation. As classical nuclear receptors for estrogens, ERα and ERβ are well-known to function in the nucleus to module transcription of specific target genes by binding to associated DNA regulatory sequences [61]. However, in addition to the transcriptional activity, a sub-population of ERα and ERβ molecules in the extranuclear pool can also attach to the cytomembrane and form signaling complexes with estrogenic ligands to initiate rapid actions within seconds or minutes [62]. Consistently, we and others reported that E2, PPT, or DPN rapidly modulates the activity dynamics of several different neural populations in an ERα- or ERβ-dependent manner [32, 33, 63, 64], suggesting potential physiological functions mediated by membrane ERα and ERβ. In supporting this view, a point mutation at the palmitoylation site of ERα (C451A-ERα), which is required for trafficking of the endogenous receptor to the plasma membrane [65], abolished membrane-bound ERα activity and impaired the neural responses of hypothalamic ERα neurons to hypoglycemia [66]. Therefore, the observed rapid responses of 5-HT^DRN^ neurons to E2, PPT, or DPN are presumably attributed to membrane-bound ERα or ERβ.

Another critical observation supporting our models is that chemogenetic activation of ERα-expressing 5-HT^DRN^ neurons in females resulted in a significant decrease in alcohol consumption without affecting sucrose intake in the DID tests. Notably, activation of ERβ^DRN^ neurons also induced similar inhibition on ethanol intake, consistent with the observation that a subpopulation of 5-HT^DRN^ neurons co-expressing ERα and ERβ. These results indicate that alcohol-induced acute stimulation of 5-HT^DRN^ neurons may serve as a defense mechanism to limit alcohol drinking. These observations align well with our findings that female mice have attenuated serotonergic responses to alcohol and more profound alcohol DID behavior. Notably, the attenuated alcohol responses in 5-HT^DRN^ neurons are at least partially attributed to estrogen/ERα/ERβ signaling as indicated by our ex vivo recording. Therefore, estrogens-mediated sex differences in the excitation of 5-HT^DRN^ neurons induced by ethanol may partially contribute to the sex dimorphism in binge-like drinking behavior in mice.

Notably, concerns about the specificity of chemogenetic activation/inhibition have been recently raised [34]. It has been shown that systemic CNO injection is converted into clozapine, a 5-HT 2A/2C receptor antagonist. CNO-converted clozapine also shows high DREADD affinity and potency. The behaviors induced by the DREADD system could be mediated by clozapine, an antipsychotic medication, instead of CNO. To address this concern, we included control *Esr1*-Cre or *Esr2*-Cre mice receiving the AAV-DIO-mCherry injection into the DRN. The potential non-specific effects induced by CNO-converted clozapine were controlled by comparing ERα-Dq^DRN^/CNO and ERα-mCherry^DRN^/CNO or ERβ-Dq^DRN^/CNO and ERβ-mCherry^DRN^/CNO.

In conclusion, we demonstrated that the DRN 5-HT system differs significantly between sexes and undergoes profound sex-specific changes during chronic alcohol DID. Furthermore, estrogens may inhibit ethanol-induced acute excitation of 5-HT^DRN^ neurons, resulting in higher levels of binge drinking in females. Notably, different neuroactive mechanisms seem to be involved in the regulatory effects of estrogens on baseline activity and ethanol-induced burst firing. Additional studies are warranted to know what intracellular channels modulate the responsiveness of 5-HT^DRN^ neurons to ethanol. Whatever the exact mechanism, the significantly lower responses of female 5-HT^DRN^ neurons to ethanol may reduce a burst of 5-HT-mediated neurotransmission during alcohol binge drinking, thus diminishing a protective mechanism for alcohol overconsumption. It could partially explain the higher binge-like drinking behavior in female mice and sex differences in human alcohol drinking behavior.

## Materials and methods

### Animals

Several transgenic mouse lines were maintained on a C57BL/6J background. These lines include *Esr1*-Cre mice (#017911, Jackson Laboratory, Bar Harbor, ME), *Esr2*-Cre mice (#30158, Jackson Laboratory), TPH2-iCreER (#016584, Jackson Laboratory), Rosa26-LSL-tdTOMATO (#007914, Jackson Laboratory), ERβ-EGFP (#030078-UCD, MMRRC at UC Davis), and ERα-ZsGreen [67]. C57BL/6J male and female mice were purchased from The Jackson Laboratory. TPH2-iCreER and Rosa26-LSL-tdTOMATO were crossed to generate TPH2-iCreER/Rosa26-LSL-tdTOMATO for electrophysiological recording. *Esr2*-Cre and Rosa26-LSL-tdTOMATO were crossed to generate *Esr2*-Cre/Rosa26-LSL-tdTOMATO for immunofluorescent staining. Mice were housed in a temperature-controlled environment at 22–24 °C on a 12-h light/dark cycle (light off at 6 pm) or a 12-h reversed light/dark cycle (light off at 10 a.m.). Unless otherwise stated, the mice were fed ad libitum with standard mouse chow (6.5% fat, #2920, Harlan-Teklad, Madison, WI) and water. Care of all animals and procedures were approved by the Pennington Biomedical Research Center (PBRC) and The University of Illinois at Chicago Institutional Animal Care and Use Committees.

### Chronic drinking in the dark (DID)

Both male and female C57BL/6J mice were subjected to 9-week-long water or ethanol DID. The 4-day DID test was performed as described previously with minor modifications [68]. Briefly, mice were randomized by body weight and individually housed in a 12-h reversed light/dark cycle room (lights off at 10 a.m. and on at 10 p.m.) for 2 weeks before behavioral testing. For the water DID group, water consumption was measured by replacing the water bottle 3 h into the dark cycle with a single-sipper tube containing only water. On the first three days (Monday, Tuesday, and Wednesday), mice were given access to the sipper tube for 2 h. On the fourth day, mice were given access to the sipper tube for 4 h. Total consumption of water over the 2-h (1st–4th days) and 4-h (4th day) period was measured in each individual. Similarly, for the ethanol DID group, ethanol consumption was measured by the ethanol DID test. Mice underwent a DID test identical to the water consumption test, except the sipper tube contained 20% ethanol in water instead of only water. The water or ethanol DID was performed on Mon–Thurs each week for 9 weeks, with no ethanol access on Fri–Sun. Sixteen hours after the last drinking session (9 AM), mice were euthanized via cardiac puncture under anesthesia. The dorsal raphe nucleus (DRN) was punched out and stored at −80 °C for further analysis.

### DREADD stimulation of ERα^DRN^ or ERβ^DRN^ neuron

Female *Esr1*-Cre or *Esr2*-Cre were stereotaxically injected with 400 nL AAV-DIO-hM3Dq-mCherry (#GVVC-AAV-130, 4.8 × 10^12^ GC/ml, Stanford Virus Core) or AAV-DIO-mCherry (5.2 × 10^12^ GC/ml, UNC Vector Core) into the DRN (4.65 mm posterior, 0 mm lateral and 3.60 mm ventral to the Bregma, based on Franklin & Paxinos Mouse Brain Atlas) at 8 weeks of age. Four weeks after the surgery, all mice were singly housed and acclimated into a 12-h reversed light/dark cycle room. Two weeks later, ethanol DID was performed as described above. Specifically, all mice were trained for 4 weeks without treatment using the EtOH DID procedure. This was done to ensure that all surgical mice exhibited stable EtOH binge drinking behavior. Following the training period, the mice underwent 1 week of saline IP injection 2 h into the dark cycle in each drinking session day for adaptation. One week after the saline injection. Clozapine N-oxide (CNO, 3 mg/kg in saline, HY-17366, MedChem Express, Monmouth Junction, NJ) was intraperitoneal (i.p.) injected 2 h into the dark cycle in each drinking session day. This protocol ensured that all mice were accustomed to the stress induced by the injection while still displaying typical EtOH drinking behavior. The investigator was blinded to the group allocation during the experiment. One week later, sucrose consumption was measured by the sucrose DID test. All the procedures were identical to the ethanol DID test, except the sipper tube contained 2% sucrose in water. After studies, all mice were perfused with 10% formalin, and the brains were collected and sliced. Sections were collected for immunohistochemistry of mCherry. Briefly, brain sections were incubated with rabbit anti-DsRed antibody (1:1000; #632496, Takara Bio., Mountain View, CA) at room temperature overnight, followed by the biotinylated donkey anti-rabbit secondary antibody (1:1000, #711-067-003, Jackson ImmunoResearch, West Grove, PA) for 1 h. Sections were then incubated in the avidin-biotin complex (1:1000, PK-6100, Vector Laboratories) and followed by 0.04% 3,3′-diaminobenzidine in 0.01% hydrogen peroxide. After dehydration through graded ethanol, the slides were immersed in xylene and cover slipped. Bright-field images were analyzed. Any mice that did not display mCherry signals in the DRN were excluded from the analysis.

Another aliquot of sections from *Esr1*-Cre mice injected with AAV-DIO-hM3Dq-mCherry was used for immunofluorescent staining of TPH. Briefly, brain sections were incubated with sheep anti-TPH antibody (1:1000, #AB1541, Millipore) at room temperature overnight, followed by the Alexa Fluor 488-conjugated donkey anti-sheep (1:500; #713-545-003, Jackson ImmunoResearch) for 2 h. Fluorescent images were obtained using a Leica DM5500 fluorescence microscope with OptiGrid structured illumination configuration.

### Real-time RT-PCR in DRN

Total mRNAs were extracted using TRIzol (#15596018, Invitrogen, Carlsbad, CA). SYBR Green quantitative PCR (qPCR) was performed, as described previously [69, 70]. Primer sequences are listed in Table 1. Results were normalized by the expression of *Gapdh* as the reference gene.

**Table 1:** qPCR primer sequences of related genes.

Gene abbreviation	Forward primer (5’–3’)	Reverse primer (5’–3’)
*Esr1*	CCCGCCTTCTACAGGTCTAAT	CTTTCTCGTTACTGCTGGACAG
*Esr2*	CTGTGATGAACTACAGTGTTCCC	CACATTTGGGCTTGCAGTCTG
*Sert*	TGGATAGTACGTTCGCAGGC	AGATGCAAGTGATGACCACGAT
*Pet1*	CTGCTGATCAACATGTACCTGC	GGAGAAACTGCCACAACTGGA
*Tph2*	GCAAGACAGCGGTAGTGTTCT	CAGTCCACGAAGATTTCGACTT
*Gapdh*	AGGTCGGTGTGAACGGATTTG	TGTAGACCATGTAGTTGAGGTCA

### Co-staining of ERα, ERβ, and TPH in the DRN

Both male and female *Esr2*-Cre/Rosa26-LSL-tdTOMATO mice were perfused with 10% formalin at 8 weeks of age. For female mice, vaginal smears were gently performed at 1 p.m. every day to determine the estrous state based on microscopic cytology, as we previously did before [63, 71]. Female mice were perfused at diestrus when circulating estrogens were high [72]. The brains were collected and sliced. Sections were collected for double staining of ERα and TPH. Briefly, brain sections were incubated with rabbit anti-ERα antibody (1:5,000; #06-935, Millipore, Burlington, MA) at room temperature overnight, followed by the Alexa Fluor 647-conjugated donkey anti-rabbit secondary antibody (1:500, #711-605-152, Jackson ImmunoResearch) for 2 h. Sections were then incubated in sheep anti-TPH antibody (1:1000, #AB1541, Millipore) overnight, followed by the Alexa Fluor 488-conjugated donkey anti-sheep (1:500; #713-545-003, Jackson ImmunoResearch) for 2 hours. Fluorescent images were obtained using a Leica DM5500 fluorescence microscope with OptiGrid structured illumination configuration.

As we described before [70, 73, 74], the images were processed and overlayed by Photoshop CS6 (San Jose, CA). The numbers of TPH (+), ERα (+), ERβ (+), TPH (+) ERα (+), TPH (+) ERβ (+), ERα (+) ERβ (+), and TPH (+) ERα (+) ERβ (+) neurons in the DRN were manually counted by blinded investigators in 5 consecutive brain sections containing the DRN, and the average neuron number per section was used to reflect the data value for that mouse. Three mice were included in each group for statistical analyses.

Similarly, double immunofluorescent staining of ERα and TPH was performed in DRN sections from a female ERβ-EGFP mouse. All staining procedures are identical except using the Alexa Fluor 594-conjugated donkey anti-sheep (1:500; #713-585-003, Jackson ImmunoResearch) as the secondary antibody for TPH.

### Electrophysiology

To investigate the sex-specific ex vivo responses of DRN neurons to the acute ethanol treatment in the brain slice recording, we used both male and female alcohol-naïve ERα-ZsGreen or TPH2-iCreER/Rosa26-LSL-tdTOMATO mice. Brain slices from female mice were collected at diestrus when circulating estrogens were high. The neurons recorded were randomly spread out the whole DRN. There were no regional differences in the recorded response.

The electrophysiological responses of identified ERα neurons in the DRN to ethanol treatment were investigated in ERα-ZsGreen mice as previously described [32]. Briefly, whole-cell patch-clamp recordings were performed on identified green fluorescent neurons in the brain slices containing DRN from ERα-ZsGreen mice. Six to 12-week-old mice were deeply anesthetized with isoflurane and transcardially perfused with an ice-cold, carbogen-saturated (95% O_2_, 5% CO_2_) sucrose-based cutting solution (pH 7.3), containing 10 mM NaCl, 25 mM NaHCO_3_, 195 mM Sucrose, 5 mM Glucose, 2.5 mM KCl, 1.25 mM NaH_2_PO_4_, 2 mM Na pyruvate, 0.5 mM CaCl_2_, 7 mM MgCl_2_. The entire brain was removed and coronally cut into slices (250 µm) with a VT1200 S vibratome (Leica). Then, the DRN-containing slices were incubated in oxygenated aCSF (adjusted to pH7.3) containing (in mM) 126 NaCl, 2.5 KCl, 2.4 CaCl_2_, 1.2 NaH_2_PO_4_, 1.2 MgCl_2_, 11.1 glucose, and 21.4 NaHCO_3_ for one hour at 34 °C.

Slices were transferred to the recording chamber and perfused at 34 °C in oxygenated aCSF at a flow rate of 1.8–2 mL/min. ZsGreen-labeled ERα^DRN^ neurons were visualized using epifluorescence and IR-DIC imaging. The intracellular solution (adjusted to pH 7.3) contained the following (in mM): 128 K gluconate, 10 KCl, 10 HEPES, 0.1 EGTA, 2 MgCl2, 0.05 Na-GTP and 0.05 Mg-ATP. Recordings were made using a MultiClamp 700B amplifier (Molecular Devices, Sunnyvale, CA, USA), sampled using Digidata 1440A, and analyzed offline with pClamp 10.3 software (Molecular Devices). Series resistance was monitored during the recording, and the values were generally <10 MΩ and were not compensated. The liquid junction potential (LJP) was +12.5 mV and was corrected after the experiment. Data was excluded if the series resistance increased dramatically during the experiment or without overshoot for the action potential. Currents were amplified, filtered at 1 kHz, and digitized at 20 kHz. The current clamp was engaged in testing neuronal firing and resting membrane potential before and after a 1 s puff of aCSF containing vehicle or ethanol (0.1, 0.5, 1, 10, or 50 mM) in the presence of synaptic cocktail inhibitors (30 μM CNQX + 30 μM D-AP5 + 50 µM bicuculline).

To study the effect of a selective ERβ agonist, diarylpropionitrile (DPN), on the activity of 5-HT neurons, a cohort of both male and female TPH2-iCreER/Rosa26-LSL-tdTOMATO mice was generated. Tamoxifen (0.2 mg/g body weight) was i.p. injected to induce expression of tdTOMATO in 5-HT neurons 4 weeks before the brain slice recording. The current clamp was engaged in testing neural firing and resting membrane potential of tdTOMATO-labeled 5-HT neurons in the DRN before and after a 1 s puff of aCSF containing a vehicle or 100 nM DPN in the presence of synaptic cocktail inhibitors (30 μM CNQX + 30 μM D-AP5 + 50 µM bicuculline).

In a different cohort of TPH2-iCreER/Rosa26-LSL-tdTOMATO mice, the brain slices were pre-incubated with aCSF containing synaptic cocktail inhibitors (30 μM CNQX + 30 μM D-AP5 + 50 µM bicuculline), 17β-estradiol (E2, 500 μM) + synaptic cocktail inhibitors, propyl pyrazole triol (PPT, 300 nM) + synaptic cocktail inhibitors, or DPN (300 nM) + synaptic cocktail inhibitors. PPT is a selective ERα agonist, while DPN is a selective ERβ agonist. The current clamp was engaged in testing neural firing and resting membrane potential before and after a 1 s puff of aCSF containing a vehicle or 1 mM ethanol in the presence of synaptic cocktail inhibitors, E2 + synaptic cocktail inhibitors, PPT + synaptic cocktail inhibitors, or DPN + synaptic cocktail inhibitors.

To assess the effect of CNO on ERα neurons, 8- to 10-week-old *Esr1*-Cre mice were injected with AAV-DIO-hM3Dq-mCherry into the DRN 2–3 weeks before recording. Brain slices were prepared and CNO was applied to the bath solution through perfusion as previously described [75]. Effects of CNO (10 µM) on membrane potential and firing frequency of mCherry-labeled ERα in the DRNA were electrophysiologically recorded.

### Statistics

The minimal sample size was predetermined by the nature of the experiments. For DID tests, 5–13 mice per group were included. For immunofluorescent quantification, 3 mice were included in each group. For electrophysiological studies, 5–38 neurons from 3 mice in each condition were included. Statistical analyses were performed using GraphPad Prism 7.0 statistics software (San Diego, CA USA). Statistical analysis methods and a number of samples were chosen based on the design of each experiment and indicated in the figure legends. The data were presented as mean ± SEM. *p* ≤ 0.05 was considered statistically significant.

### Supplementary information

Supplemental Material

## Data Availability

The authors confirm that the data supporting the findings of this study are available within the article and/or its supplementary materials.
