# Supplementary material for: Estrogen signaling in the dorsal raphe regulates binge-like drinking in mice
Source: Transl Psychiatry. 2024 Feb 27;14:122. doi: 10.1038/s41398-024-02821-2 (PMC10899193; doi:10.1038/s41398-024-02821-2)
Supplement: Supplementary file 1 — Supplemental Material [file 41398_2024_2821_MOESM1_ESM.docx]

**Supplemental figure 1**


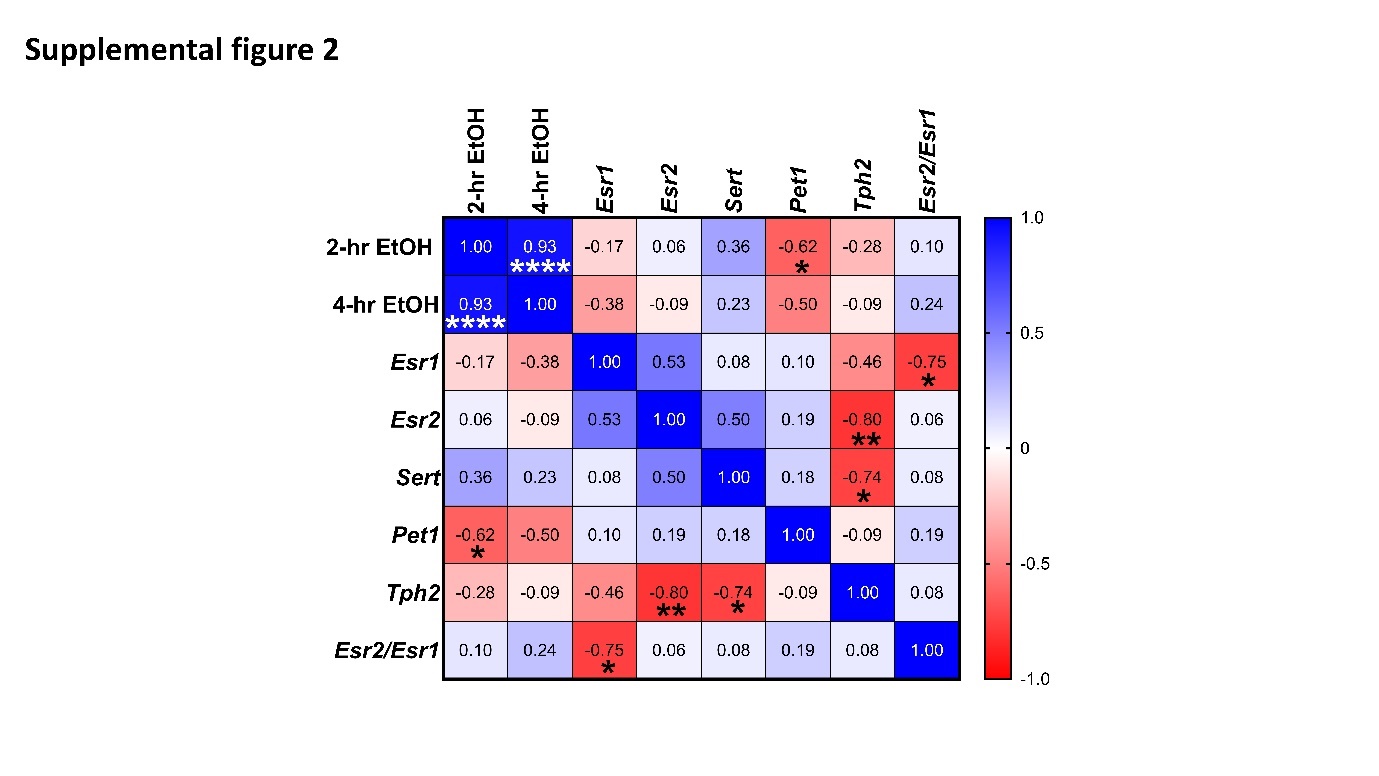


**Supplemental fig. 1 (related to Fig. 1). Correlation of alcohol consumption and individual expression levels.** Two-tailed Pearson’s correlation coefficient analysis of average 2-hr or 4-hr alcohol consumption and mRNA expression of *Esr1, Esr2, Sert, Pet1, Tph2, and Esr2/Esr1* in mice (6 males and 4 females). The blue color represents a positive correlation, and the red represents a negative one. *p<0.05, **p<0.01, ****p<0.0001 indicate significant correlation.

**Supplemental figure 2**


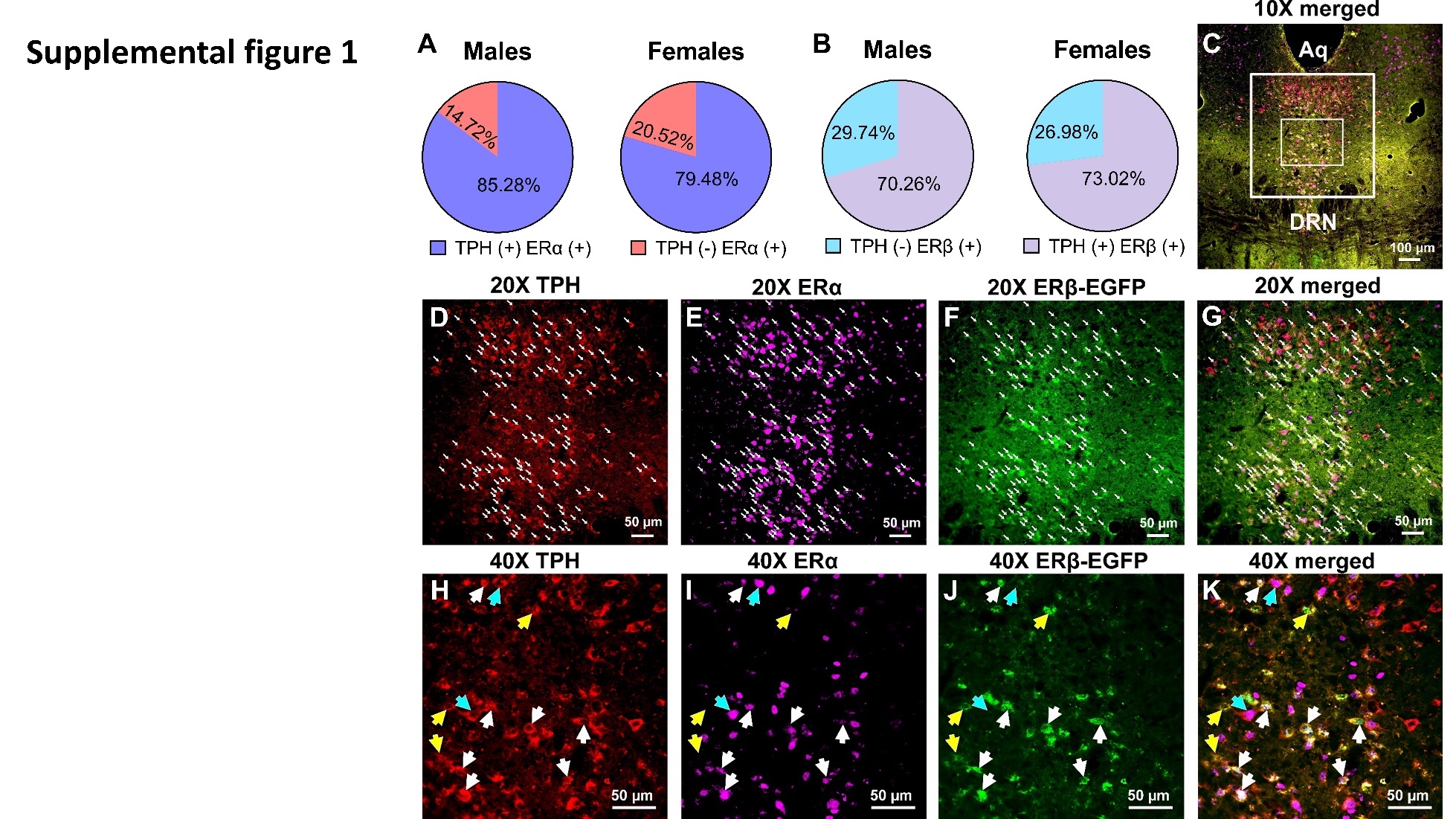


**Supplemental fig. 2 (related to Fig. 2). A large portion of ERα^DRN^ or ERβ^DRN^ neurons are serotonergic neurons.** (A-B) Percentage of TPH positivity in ERα^DRN^ (A) or ERβ^DRN^ (B) neurons from male and female ERβ-Cre/Rosa26-tdTOMATO mice. (C-K) Low (C) and high magnification of TPH (red, D and H), ERα (purple, E and I), ERβ-EGFP (green, F and J), and merged (G and K) in the DRN of female ERβ–EGFP mouse. The white square represents a 20X or 40X enlarged area. White arrows point to triple positive TPH (+) ERα (+) ERβ (+) neurons. Yellow arrows point to dual-positive TPH (+) ERβ (+) neurons. Cyan arrows point to dual-positive TPH (+) ERα (+) neurons.

**Supplemental figure 3**


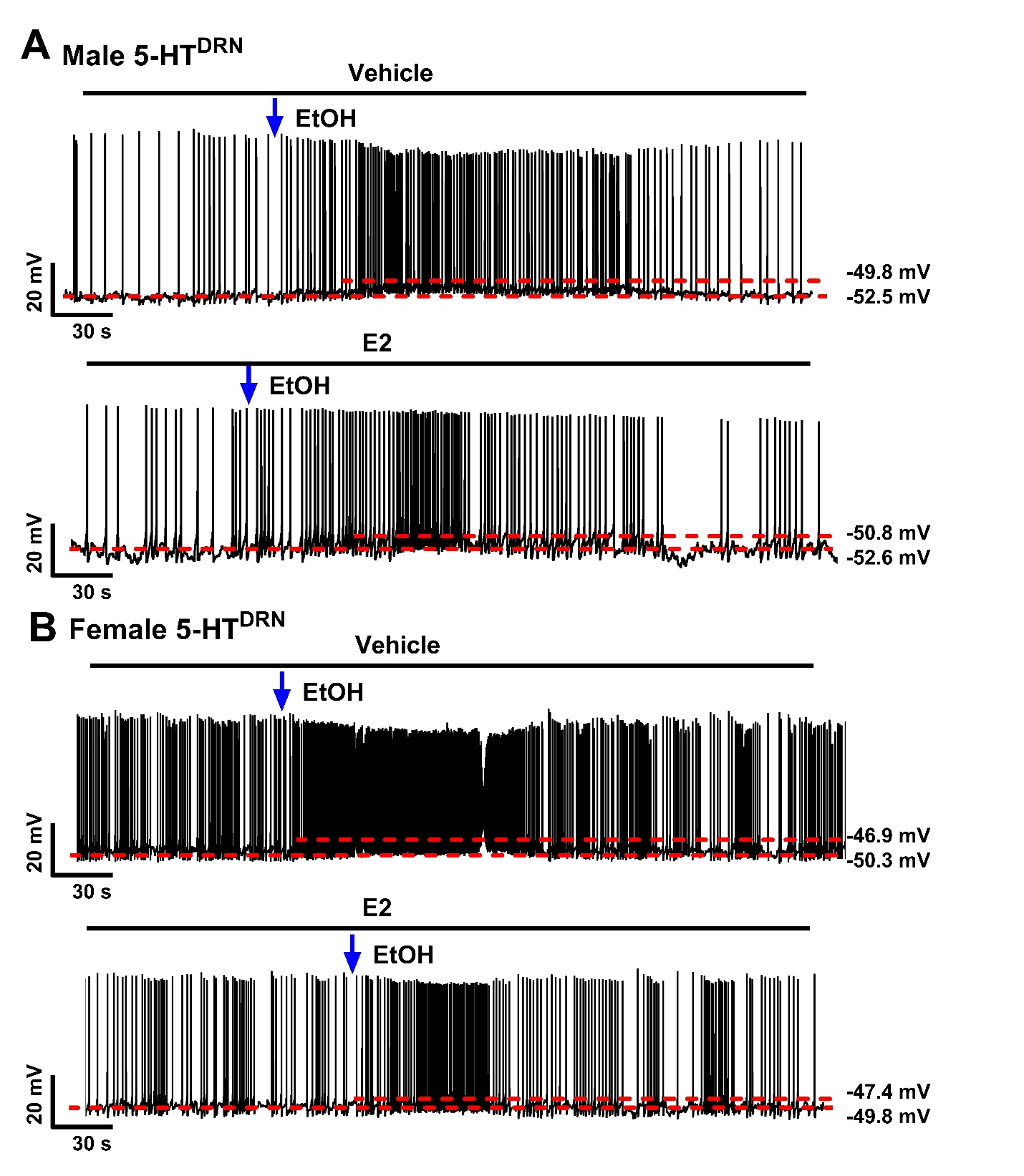


**Supplemental fig. 3 (related to Fig. 4). Representative traces of 5-HT^DRN^ neurons after EtOH treatment in the presence of vehicle or E2.**

**Supplemental figure 4**


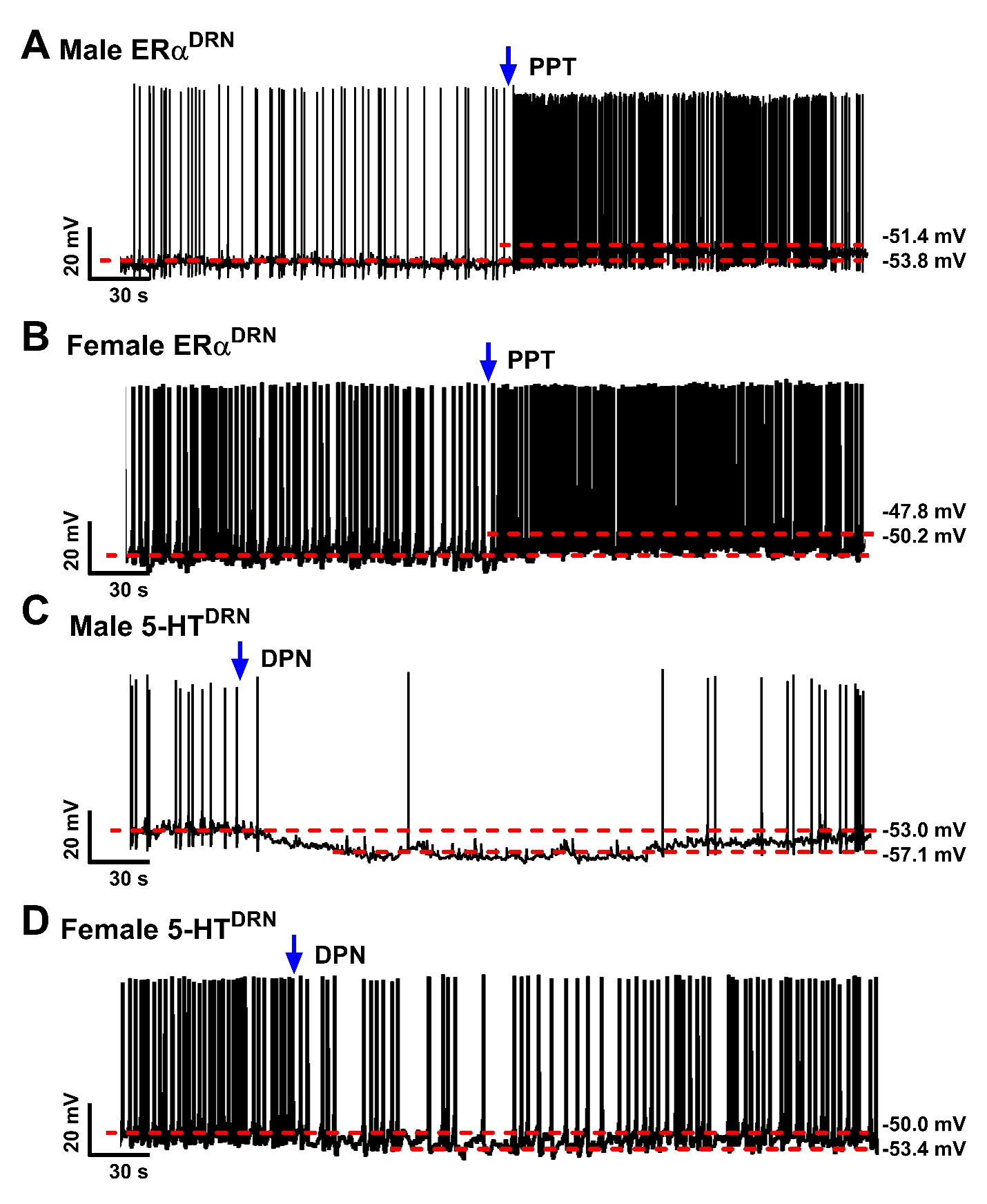


**Supplemental fig. 4 (related to Fig. 5). Representative traces of ERα^DRN^ or 5-HT^DRN^ neurons following treatment with the ERα agonist PPT or the ERβ agonist DPN.**
